# Reports of the Surgeon-General

**Published:** 1879-02

**Authors:** 


### Books Reviewed.

Annual Reports of the Supervising Surgeon-General of the Marine Hospital Service of the United States, for the Fiscal years 1876 and 1877. By JOHN M. WOODWORTH, M. D., Surgeon-General, Washington.

These reports are full and complete in detail and have invoked a vast amount of clerical labor. The tables show that a large number of the Seamen coming under the care of the Marine Hospital Service are annually permanently unfitted for duty as Seamen. In 1876 over three hundred cases of Phthisis; One hundred diseases of the Calculatory System; Seventy-five of Bright's disease; Forty-eight cases of Hernia; Eighty-eight cases of Paralysis, and two thousand cases of Syphilis are recorded.

The work is highly creditable to the Surgeon-General, indicating the extent and character of the labor in this department of the Marine Service.

